# Small RNA promotes negative feedback of the master virulence regulator PhoP by repressing the PhoQ sensor enhancer UgtL in acidic pH

**DOI:** 10.1128/msphere.00720-25

**Published:** 2025-12-09

**Authors:** Michelle D. Prophete, Alexander Mabel, Payton Bowman, Hubert Salvail

**Affiliations:** 1 Division of Immunity and Pathogenesis, Burnett School of Biomedical Sciences, University of Central Florida, College of Medicine 124507, Orlando, Florida, USA; US Food and Drug Administration, Silver Spring, Maryland, USA

**Keywords:** PinT, PhoP/PhoQ, signal transduction, UgtL, sRNA, two-component systems, acidic pH, virulence program

## Abstract

Signal transduction allows bacterial pathogens to sense the host environment and regulate gene expression accordingly for adaptation and survival. While the success of infection largely depends on the timely induction of virulence genes, the activity of the regulatory pathways controlling their expression must be tightly regulated for pathogens to cause disease. Here, we establish that a small RNA (sRNA) promotes the negative feedback control of a master virulence regulator in *Salmonella enterica* serovar Typhimurium (*S*. Typhimurium) by repressing a signaling protein essential for its induction in response to an intracellular cue. We show that the virulence regulatory PhoP/PhoQ pathway is inhibited by the PhoP-activated sRNA PinT in mildly acidic pH, an infection-relevant condition encountered by *S*. Typhimurium inside macrophages. PinT directly represses the translation of *ugtL* mRNA, which encodes the PhoP activator UgtL. This negative feedback regulation reduces PhoP activity, thereby decreasing the expression of PhoP-activated virulence genes like *pagC*. PinT-mediated repression of *ugtL* is predicted to be conserved in *Salmonella enterica*, but not in the nonpathogenic species *Salmonella bongori*, thus suggesting that the regulation is relevant for virulence. Our findings uncover how pathogens achieve proper levels of induction of their virulence programs through the post-transcriptional negative feedback regulation of factors enhancing the signaling activity of virulence pathways.

**IMPORTANCE:**

To cause disease, pathogens must express their virulence genes at the right time and in proper levels. Here, we establish that a small RNA (sRNA) restricts the activation of a regulator critical for the virulence of *Salmonella enterica* serovar Typhimurium (*S.* Typhimurium). We show that the sRNA PinT inhibits the activity of the master virulence regulator PhoP by repressing its activator UgtL through a direct interaction with *ugtL* mRNA. This regulation reduces the expression of PhoP-activated genes. Because PhoP activates PinT and UgtL, the three regulators form a negative feedback loop. That the PinT-mediated repression of *ugtL* is predicted to occur in *Salmonella enterica* but not in the nonpathogenic species *S.* bongori suggests it may be a key virulence determinant. Our results unveil a novel layer of fine-tuning of PhoP activity ensuring that *S.* Typhimurium induces the proper level of its virulence program in response to an infection-relevant stress condition.

## INTRODUCTION

Pathogens’ ability to cause disease depends on the timely induction of virulence programs in response to stress conditions experienced inside the host (1). Sustained or excess expression of virulence genes can impede the success of infection by interfering with the normal cellular functions of the pathogen or triggering the host immune response (2). To maintain proper levels of virulence program induction, the regulon of virulence regulatory pathways usually comprises genes encoding negative regulators, restraining their activity (3–5). Here, we identify a post-transcriptional negative feedback regulation allowing the facultative intracellular pathogen *Salmonella enterica* serovar Typhimurium (*S*. Typhimurium) to fine-tune the activity of a master virulence regulator in response to an infection-relevant stress condition.

The two-component system PhoP/PhoQ is a major regulator of *S*. Typhimurium virulence (6, 7). In response to various signals, including mildly acidic pH and low magnesium (Mg^2+^), the sensor kinase PhoQ autophosphorylates and then transfers a phosphate to the response regulator PhoP (PhoP-P), thus leading to its activation. PhoP-P regulates the expression of a large set of genes by directly binding to their promoter to alter their transcription (8). The PhoP regulon comprises genes involved in various cellular activities, including virulence, Mg^2+^ transport, cell surface modification, and antimicrobial resistance (9). PhoP also promotes the transcription of the small RNA (sRNA) PinT upon infection of mammalian cells (10). PinT is an 80-nt-long Hfq-dependent sRNA that coordinates the transition between the SPI-1 invasion and SPI-2 intracellular gene expression programs by post-transcriptionally regulating the expression of multiple genes encoding effectors and regulators associated with both programs through direct interaction with their mRNA. It represses the expression of the *sopE* and *sopE2* genes encoding effectors promoting invasion of epithelial cells by stimulating membrane ruffling (10) and the translation of *hilA* and *rtsA* mRNAs specifying two master regulators of SPI-1 genes (11). It also delays the induction of SPI-2 genes by inhibiting the expression of CRP (10), which promotes their transcription, and represses the translation of *steC*, which encodes a secreted SPI-2 effector inducing actin rearrangement inside epithelial cells (12).

PhoP activation in mildly acidic pH conditions is necessary for *S*. Typhimurium’s survival inside macrophages (13). This activation requires the *ugtL* gene (14). UgtL is an inner membrane protein that interacts with PhoQ to promote its autophosphorylation, thus leading to increased amounts of PhoP-P. While the induction of PhoP virulence program in response to mildly acidic pH is required for *S*. Typhimurium’s survival inside macrophages (13), PhoP activity must be tightly regulated as constitutive *phoP* expression attenuates virulence (15, 16). This is reflected by the *ugtL* gene being subjected to multiple layers of regulation in mildly acidic pH conditions in addition to being transcriptionally activated by PhoP (14, 17). First, the regulator SsrB promotes *ugtL* transcription by antagonizing H-NS silencing (18). Second, *ugtL* mRNA requires translational activation by the RNA chaperone CspC, whose regulation is necessary for the PhoP virulence program to be induced in mildly acidic pH conditions (19). Finally, the small 34-amino acid UgtS protein interacts with UgtL to prevent it from activating PhoQ, thereby resulting in reduced PhoP activation (4). UgtS is encoded with UgtL in an operon (*ugtSugtL*) that is transcribed via two transcription start sites (TSSs) specifying two distinct *ugtSugtL* mRNAs with *ugtL*’s 5′ leader regions of 182 and 171 nts in length and only the longer transcript (*ugtSugtL_-182_*) harboring the *ugtS* Shine-Dalgarno sequence and thus allowing *ugtS* translation. The two transcript isoforms describe distinct expression profiles when *S*. Typhimurium is inside macrophages, thereby allowing the bacteria to time the induction of the PhoP regulon by controlling the amounts of UgtS and UgtL proteins, and then PhoP activity.

**Fig 1:**
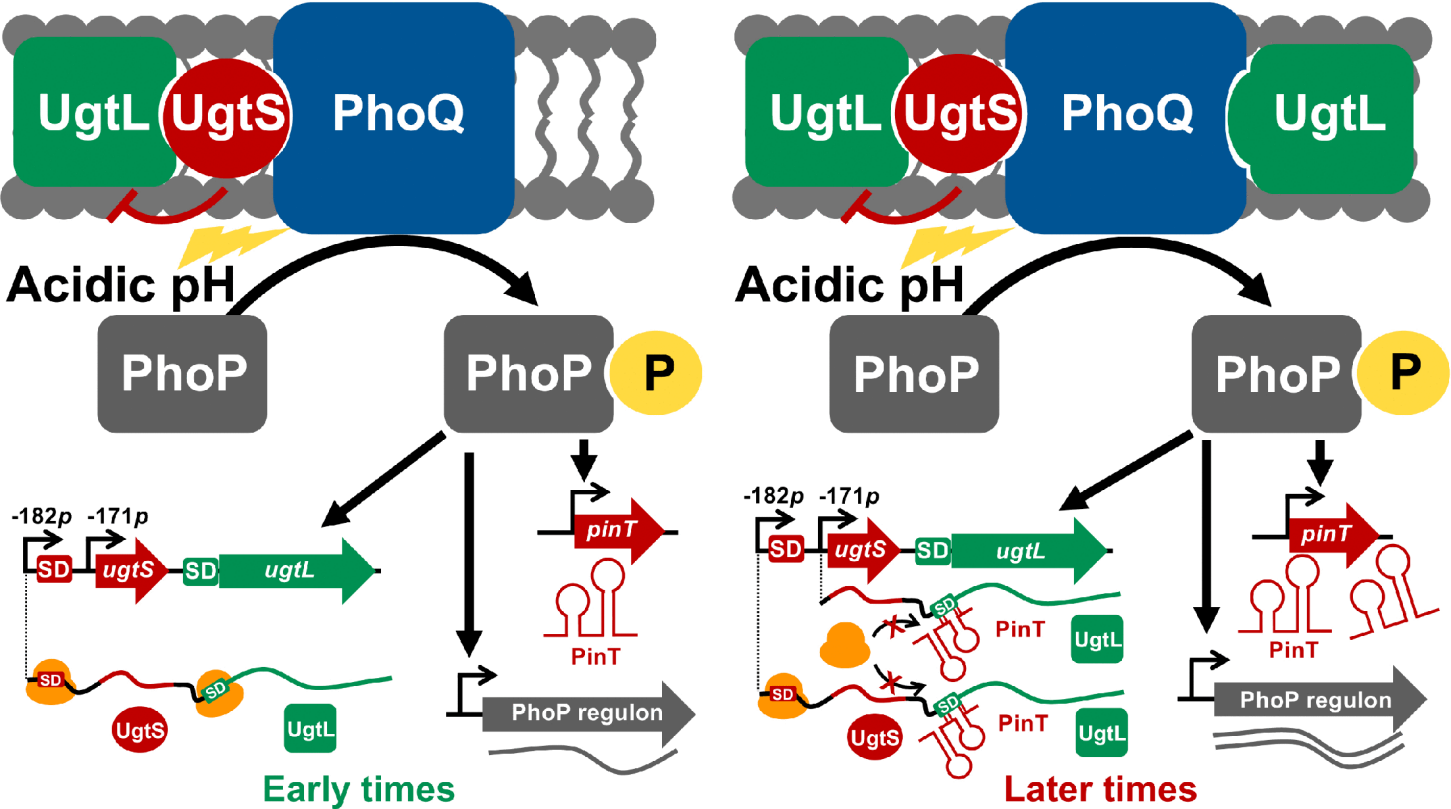
The sRNA PinT promotes negative feedback regulation of the master virulence regulator PhoP in mildly acidic pH conditions. At early time points of growth in mildly acidic pH conditions (*left*), PhoQ is autophosphorylated and then promotes the phosphorylation of the response regulator PhoP. PhoP-P activates *ugtSugtL* transcription from the −182 p TSS, resulting in the production of UgtL protein whose action of promoting PhoQ autophosphorylation is antagonized by the small protein UgtS, as previously established (4). This results in low levels of UgtL-mediated activation of PhoQ, and therefore of PhoP regulon induction (Fig. 2C and 4B and E), including the *pinT* gene (Fig. 2A) that specifies the sRNA PinT. At later time points of growth in mildly acidic pH conditions (*right*), PhoP-P activates *ugtSugtL* transcription from both −182 p and −171 p TSSs (4)*,* resulting in increased synthesis of UgtL protein that enhances PhoQ autophosphorylation and then promotes further induction of the PhoP regulon (14) (including the *pinT* gene) (Fig. 2A), the regulatory activity of which is under the negative control of UgtS (4). PinT inhibits UgtL expression (Fig. 2B and C) by repressing the translation of *ugtSugtL_-182_* and *ugtSugtL_-171_* mRNAs (Fig. 3D), which results in reduced induction of the PhoP regulon (Fig. 4E). PinT is part of a negative feedback loop with the regulator PhoP that, on the one hand, promotes the production of UgtL that enhances its activity, and on the other hand, the expression of PinT that reduces UgtL production, followed by PhoP activity.

We now report that the PhoP-dependent sRNA PinT provides an additional layer of *ugtL* regulation by promoting negative feedback control of PhoP activity by repressing UgtL (Fig. 1). We establish that PinT represses *ugtL* translation through a direct interaction with its mRNA. This regulation reduces the induction of PhoP-activated genes at later time points of growth in mildly acidic pH conditions, thereby providing a negative feedback regulation of PhoP activity allowing *S*. Typhimurium to achieve optimal levels of induction of its virulence program. PinT-*ugtL* interaction is conserved in *Salmonella enterica* (*S. enterica*), but not in the nonpathogenic species *Salmonella bongori* (*S. bongori*), thus suggesting the PinT-mediated negative feedback regulation of PhoP activity to be relevant for infection.

## RESULTS

### PinT represses UgtL in mildly acidic pH conditions

**Fig 2:**
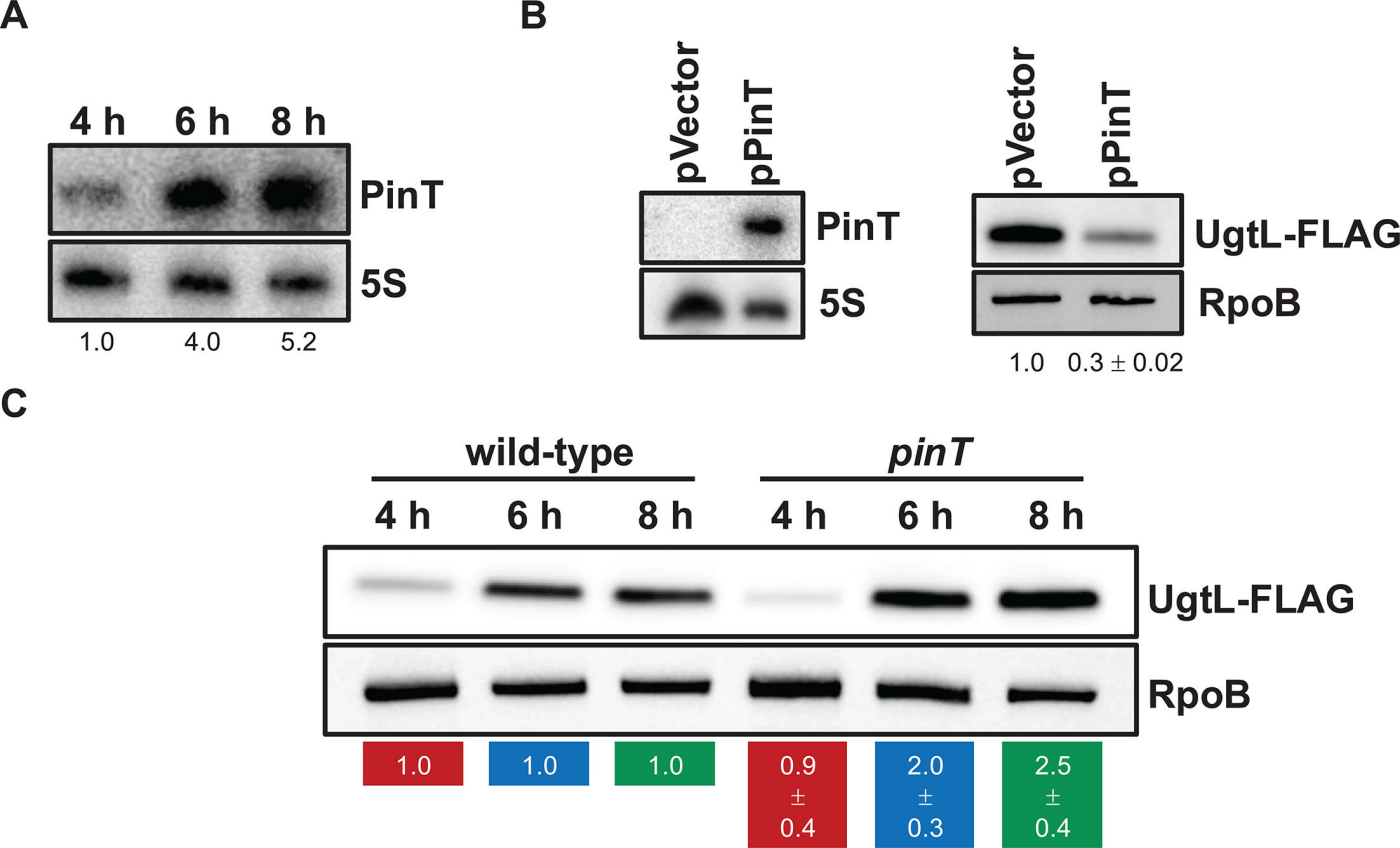
PinT represses UgtL in mildly acidic pH conditions. (**A**) Northern blot analysis of PinT abundance from total RNA extracts of wild-type *S*. Typhimurium grown in N-minimal acidic pH (pH 4.9, 1 mM MgCl_2_) medium at the indicated time points. Samples were analyzed with DNA probes complementary to PinT (HUSA404) and 5S (HUSA405). Numbers below the blot indicate PinT levels relative to the 4 h time point. (**B**, *left*) Northern blot analysis of PinT abundance from total RNA extracts of *ugtL-FLAG pinT* (MIPR004) *S*. Typhimurium harboring plasmid pPinT or pVector (empty pUHE-21 vector) grown in N-minimal acidic pH (pH 4.9, 1 mM MgCl_2_) medium supplemented with 1 mM IPTG for 6 h. Samples were analyzed with DNA probes complementary to PinT (HUSA404) and 5S (HUSA405). (B, *right*) Western blot analysis of extracts from *ugtL-FLAG pinT* (MIPR004) *S*. Typhimurium harboring plasmid pPinT or pVector (empty pUHE-21 vector) grown in N-minimal acidic pH medium (pH 4.9, 1 mM MgCl_2_) supplemented with 1 mM IPTG for 6 h. Samples were analyzed with antibodies directed to the FLAG epitope or the RpoB protein. Numbers below the blot indicate UgtL-FLAG levels relative to the pVector sample. Data are representative of two independent experiments (mean ± standard deviation), which gave similar results. (**C**) Western blot analysis of extracts from *ugtL-FLAG* (HS1189) and *ugtL-FLAG pinT* (MIPR004) *S*. Typhimurium grown in N-minimal acidic pH medium (pH 4.9, 1 mM MgCl_2_) for the indicated time points. Samples were analyzed with antibodies directed to the FLAG epitope or the RpoB protein. Numbers below the blot indicate UgtL-FLAG levels in the *pinT* background relative to the wild-type background for a given time point depicted by color (red for 4 h; blue for 6 h; green for 8 h). Data are representative of two independent experiments (mean ± standard deviation), which gave similar results.

The *ugtL* mRNA was identified as a potential target of PinT sRNA in three independent high-throughput RNA interactome studies (12, 20, 21). This suggests that *ugtL* expression, and therefore UgtL’s regulatory action of activating PhoP, is subjected to post-transcriptional feedback control by PinT (PhoP activates *ugtL* and *pinT,* and PinT, in turn, regulates *ugtL* and therefore UgtL-mediated activation of PhoP). We explored this possibility by first assessing the effect of heterologous *pinT* expression on UgtL amounts in mildly acidic pH conditions, an infection-relevant condition that *S*. Typhimurium experiences inside macrophages (22, 23), leading to PhoP activation (13). PinT levels were low at 4 h of growth (late exponential phase; Fig. S1) in mildly acidic pH conditions and increased 4-fold at 6 h (early stationary phase) and 5-fold at 8 h (mid-stationary phase), thereby indicating that the sRNA is expressed under this PhoP-inducing condition (Fig. 2A). A *pinT* mutant strain specifying a UgtL-FLAG epitope exhibited a 3-fold decrease of UgtL abundance at 6 h in mildly acidic pH conditions when carrying a plasmid with *pinT* under the control of a heterologous promoter (Fig. 2B, *left*) as compared to the same strain harboring an empty vector (Fig. 2B, *right*). A similar level of PinT-mediated repression of UgtL was observed in low Mg^2+^, another condition promoting PhoP activation (Fig. S2). This indicates that PinT is a negative regulator of UgtL regardless of the PhoP-inducing condition. The amounts of UgtL proteins were 2- and 2.5-fold higher in the *pinT* mutant strain as compared to wild-type *S*. Typhimurium at 6 and 8 h time points, respectively, in mildly acidic pH conditions, but similar between both strains at 4 h (Fig. 2C). This is consistent with PinT being a repressor of UgtL (Fig. 2B, *right*) and showing maximal expression at 6 and 8 h (Fig. 2A).

The data in this section establish that PinT is a PhoP-activated negative regulator of UgtL and then raise the possibility of the sRNA mediating negative feedback control of PhoP virulence program induction.

### PinT directly represses *ugtL* translation

**Fig 3:**
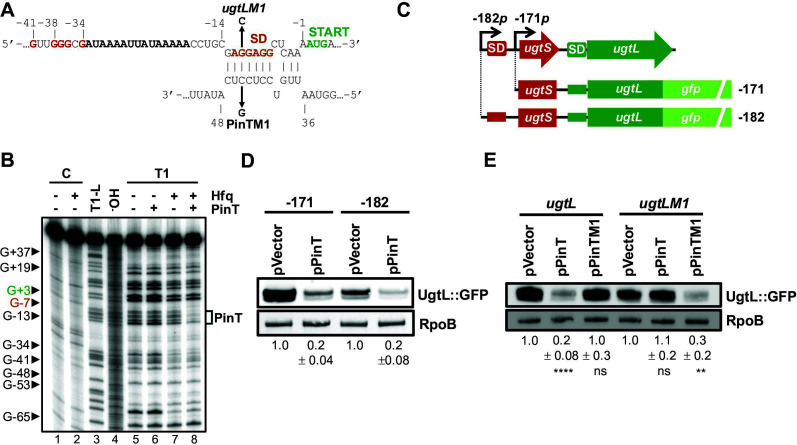
PinT directly represses *ugtL* translation. (**A**) *In silico* prediction of PinT-*ugtL* interaction. The prediction was performed using the IntaRNA program (26). The complementary PinTM1 and *ugtLM1* mutations used in the experiment described in (**E**) are indicated. Positions relative to the *ugtL* start codon and PinT TSS are marked. The G residues protected upon Hfq addition (**B**) are marked in red, and the potential AU-rich Hfq-binding site is indicated in bold. SD, Shine-Dalgarno sequence. (**B**) Enzymatic probing analysis of PinT-*ugtL* interaction. 5′-^32^P-radiolabeled *ugtL* RNA (nucleotides −171 to + 66 relative to AUG start codon) was incubated in the presence or absence of PinT (1 µM final) and with or without Hfq (500 µM final) before the addition of RNase T1. C, no reaction; T1-L, RNase T1 ladder (G nucleotides); ^-^OH, alkaline digestion (cleaves after each nucleotide); T1, partial digestion with RNase T1 (cleaves single-stranded G nucleotides). Some G residues are marked (positions relative to the *ugtL* AUG start codon). Nucleotides composing the Shine-Dalgarno sequence and AUG start codon are marked in orange and green, respectively. A version of the figure with lower contrast is provided in Fig. S6. (**C**) Schematic of the *ugtSugtL* locus and of the *ugtL* GFP fusions used in the experiments described in (**D and E**). SD, Shine-Dalgarno sequence (boxed in red for *ugtS* and in green for *ugtL*). The −182 p and −171 p (positions relative to *ugtL* ATG start codon) TSSs are indicated with arrows. (**D**) Western blot analysis of crude extracts from *pinT* (HS1440) *S*. Typhimurium harboring plasmid pPinT or pVector (empty pUHE-21 vector) and pXG10sf-*ugtL-182* or pXG10sf-*ugtL-171*, grown in N-minimal acidic pH (pH 4.9, 1 mM MgCl_2_) medium supplemented with 1 mM IPTG for 6 h. Samples were analyzed with antibodies directed to the GFP or RpoB proteins. Numbers below the blot indicate UgtL::GFP amounts relative to the pVector sample for −182 and −171 GFP fusions. Data are representative of two independent experiments (mean ± standard deviation), which gave similar results. (**E**) Western blot analysis of crude extracts from *pinT* (HS1440) *S*. Typhimurium harboring pPinT, pPinTM1 or pVector (empty pUHE-21 vector), and pXG10sf-*ugtL-171* or pXG10sf-*ugtLM1-171*, grown in N-minimal acidic pH (pH 4.9, 1 mM MgCl_2_) medium supplemented with 1 mM IPTG for 6 h. Samples were analyzed with antibodies directed to the GFP or RpoB proteins. Numbers below the blot indicate UgtL::GFP amounts relative to the pVector sample for *ugtL-171* and *ugtLM1-171* GFP fusions. Data are representative of three independent experiments (mean ± standard deviation), which gave similar results. **P < 0.01; **** *P* < 0.0001; ns, not significant (*P* > 0.05), two-tailed unpaired *t* test with pPinT or pPinTM1 vs pVector for *ugtL-171* and *ugtLM1-171* reporter constructs.

Based on previous reports suggesting that PinT interacts with *ugtL* mRNA (12, 20, 21) and our data showing that the sRNA reduces UgtL abundance in mildly acidic pH conditions (Fig. 2B and C), we evaluated the possibility of PinT base pairing with *ugtL* mRNA to inhibit its translation and/or promote its degradation, as it has been reported for the other characterized mRNA targets of the sRNA (10, 12). *In silico* base*-*pairing predictions identified a potential interaction in which PinT base-pairs with the *ugtL* Shine-Dalgarno sequence (Fig. 3A), consistent with the sRNA reducing UgtL protein amounts (Fig. 2B and C), possibly by preventing the ribosome from initiating *ugtL* translation. We first validated the predicted PinT-*ugtL* interaction by using enzymatic probing analysis. A 5′-^32^P-labeled RNA construct of *ugtL* harboring the entire 171-nt-long 5′ leader of the *ugtSugtL_-171_* transcript plus 66 nucleotides from *ugtL* open reading frame (ORF) was protected from RNase T1 (cuts single-stranded guanine residues) digestion in the presence of *in vitro*-transcribed PinT and purified Hfq protein (Fig. 3B; compare lanes 7 and 8) in the region determined to be complementary to the sRNA (G-13 to G-7). (Fig. 3A). No cleavage protection was observed in this region in the presence of PinT only (compare lanes 5 and 6), indicating that Hfq is required for PinT base pairing with *ugtL in vitro*. The residues G-34, G-36, G-37, G-38, and G-41 were protected from RNase T1 cleavage in the presence of Hfq (compare lanes 5 and 7), probably as a result of Hfq binding to the region immediately downstream of these residues (−33 to −19) that is rich in adenine and uridine, a common feature of Hfq-binding sites (24, 25). These data provide an *in vitro* validation of the *in silico* PinT-*ugtL* interaction prediction (Fig. 3A).

PinT may reduce UgtL abundance by inhibiting *ugtL* translation through base pairing with its mRNA, as predicted (Fig. 3A) and established by enzymatic probing (Fig. 3B), and/or by reducing PhoP-dependent transcription of *ugtL*, either as a consequence of the translational repression of *ugtL* or the regulation of another mRNA target. To solely assess the post-transcriptional effect of PinT on *ugtL*, plasmid-based translational fusions of *ugtL* from either the −171 or −182 transcription site to the reporter gene *gfp* under the control of a PhoP-independent constitutive promoter were generated (Fig. 3C), and the effect of PinT heterologous expression was assessed in mildly acidic pH conditions. Both −171 and −182 fusions were repressed by PinT (Fig. 3D), recapitulating the decrease of UgtL-FLAG encoded from its normal chromosomal location by the sRNA (Fig. 2B) and in agreement with the prediction that it interacts with *ugtL*’s Shine-Dalgarno sequence that is shared by both *ugtSugtL_-182_* and *ugtSugtL_-171_* transcripts.

To provide *in vivo* demonstration of PinT inhibiting *ugtL* translation through direct base pairing, we introduced single complementary mutations in plasmid-encoded PinT (PinTM1) and *ugtL-gfp* (*ugtLM1*) fusion to weaken the predicted PinT-*ugtL* duplex (Fig. 3A) and then examined the ability of PinT and PinTM1 to repress the *ugtL-gfp* and *ugtLM1-gfp* fusions. PinT and PinTM1 accumulated to similar levels when heterologously expressed in mildly acidic pH conditions, indicating that the M1 mutation does not affect PinT stability and/or transcription (Fig. S3). Expression of PinT decreased UgtL::GFP amounts fivefold (Fig. 3E) in the strain carrying the *ugtL-gfp* fusion, in agreement with PinT repressing the expression of endogenous UgtL-FLAG protein in mildly acidic pH conditions (Fig. 2B and C). PinTM1 failed to reduce UgtL::GFP abundance when expressed in the strain harboring the *ugtL-gfp* fusion, but decreased UgtL::GFP amounts threefold in the *ugtLM1-gfp* strain with the *ugtLM1* mutation complementary to PinTM1. PinT did not affect UgtL::GFP levels in the *ugtLM1-gfp* strain, consistent with the sRNA having reduced complementary to *ugtLM1* (Fig. 3A). These data demonstrate that PinT directly represses *ugtL* translation *in vivo*.

Taken together, these results show that PinT reduces UgtL amounts in mildly acidic pH conditions by base pairing with *ugtL* mRNA to repress its translation. Because UgtL activates PhoP (14), the repression of *ugtL* by PinT likely reduces PhoP activity, and therefore the induction of the PhoP regulon, including the *ugtL* gene.

### PinT reduces PhoP activity in mildly acidic pH conditions

**Fig 4:**
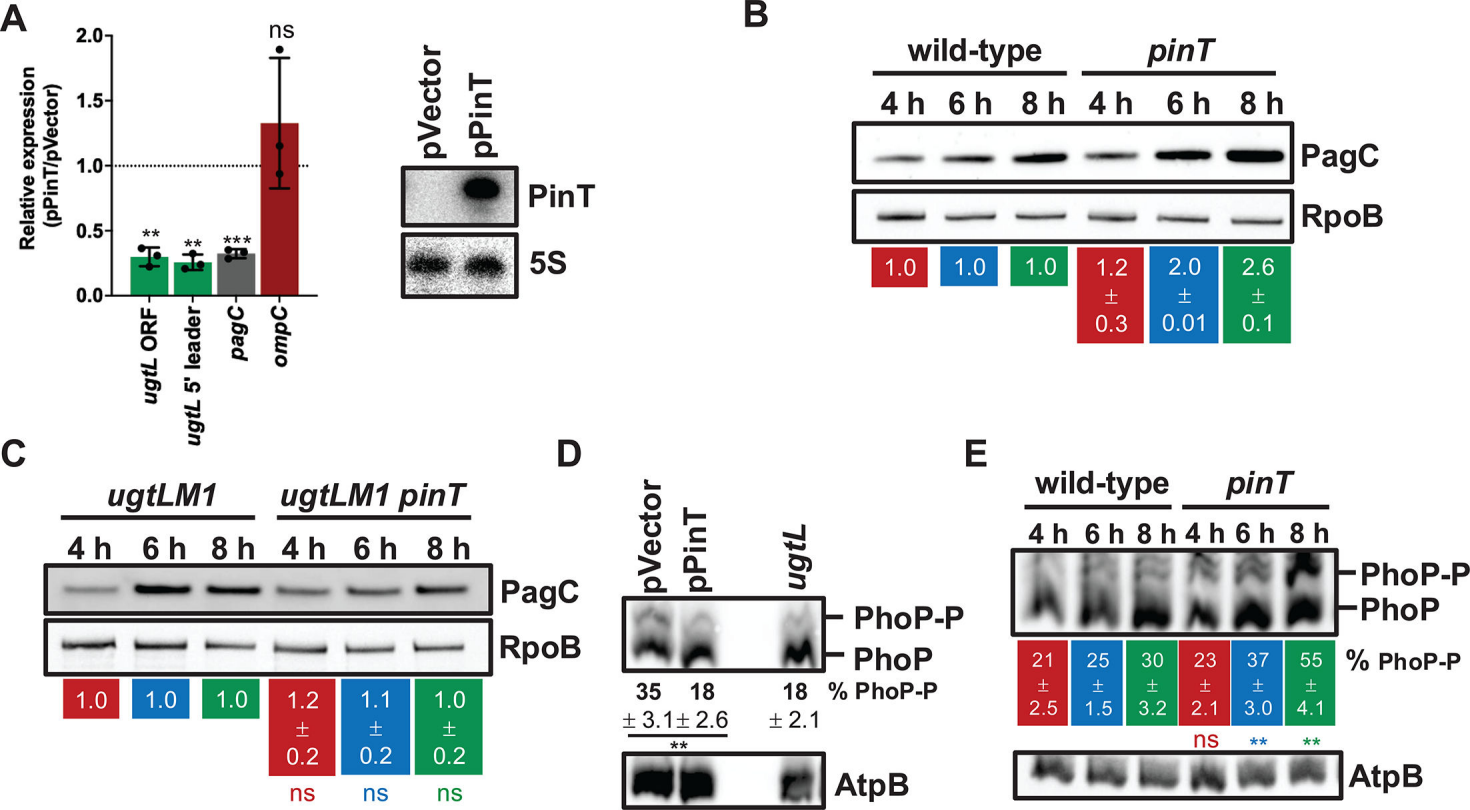
PinT reduces PhoP activity. (**A**, *left*) qRT-PCR analysis of the abundance of *ugtL*, *ugtL* 5′ leader, *pagC,* and *ompC* transcripts in *pinT* (HS1440) *S*. Typhimurium harboring plasmid pPinT or pVector grown for 6 h in N-minimal acidic pH (pH 4.9, 1 mM MgCl_2_) medium supplemented with 1 mM IPTG. Transcript abundance values were normalized to the levels of 16S ribosomal RNA. The mean and standard deviation (SD) from three independent experiments are shown. **P < 0.01, *** *P* < 0.001; ns, not significant (*P* > 0.05), one sample t test with theoretical mean of 1. (**A**, *right*) Northern blot analysis of PinT abundance from total RNA extracts of *pinT* (HS1440) *S*. Typhimurium harboring plasmid pPinT or pVector grown for 6 h in N-minimal acidic pH (pH 4.9, 1 mM MgCl_2_) medium supplemented with 1 mM IPTG. Samples were analyzed with DNA probes complementary to PinT (HUSA404) and 5S (HUSA405). (**B**) Western blot analysis of crude extracts from wild-type (14028s) and *pinT* (HS1440) *S*. Typhimurium grown in N-minimal acidic pH (pH 4.9, 1 mM MgCl_2_) medium at the indicated time points. Samples were analyzed with antibodies directed to the PagC or RpoB proteins. Numbers below the blot indicate PagC levels in the *pinT* background relative to the wild-type background for a given time point depicted by color (red for 4 h; blue for 6 h; green for 8 h). Data are representative of two independent experiments (mean ± standard deviation), which gave similar results. (**C**) Western blot analysis of crude extracts from *ugtLM1* (AMPB002) and *ugtLM1 pinT* (AMPB003) *S*. Typhimurium grown in N-minimal acidic pH (pH 4.9, 1 mM MgCl_2_) medium at the indicated time points. Samples were analyzed with antibodies directed to the PagC or RpoB proteins. Numbers below the blot indicate PagC levels in the *ugtLM1 pinT* background relative to the *ugtLM1* background for a given time point depicted by color (red for 4 h; blue for 6 h; green for 8 h). Data are representative of three independent experiments (mean ± standard deviation), which gave similar results. ns, not significant (*P* > 0.05), two-tailed unpaired *t* test with *ugtLM1 pinT* vs *ugtLM1* for each time point. (**D**) Phos-tag Western blot analysis of crude extracts obtained from *pinT* (HS1440) harboring plasmid pPinT or pVector (empty pUHE-21 vector) and *ugtL* (HS1547) *S*. Typhimurium grown for 6 h in N-minimal acidic pH (pH 4.9, 1 mM MgCl_2_) medium supplemented with 1 mM IPTG. Samples were analyzed with antibodies directed to the PhoP or AtpB proteins. The percentage of PhoP-P for each sample is indicated below the blot. Data are representative of three independent experiments (mean ± standard deviation), which gave similar results. **P < 0.01, two-tailed unpaired *t* test with pVector vs pPinT. Lane 3 was left intentionally empty. (**E**) Phos-tag Western blot analysis of crude extracts obtained from wild-type and *pinT* (HS1440) *S*. Typhimurium grown in N-minimal acidic pH (pH 4.9, 1 mM MgCl_2_) medium at the indicated time points. Samples were analyzed with antibodies directed to the PhoP or AtpB proteins. The percentage of PhoP-P for each sample is indicated below the blot for each time point depicted by color (red for 4 h; blue for 6 h; green for 8 h). Data are representative of three independent experiments (mean ± standard deviation), which gave similar results. **P < 0.01, ns, not significant (*P* > 0.05), two-tailed unpaired *t* test with *pinT* vs wild-type for each time point.

Because UgtL is required for PhoP activation in mildly acidic pH conditions (14), we reasoned the PinT-mediated repression of *ugtL* would decrease PhoP activity. We explored this possibility by monitoring the mRNA abundance of PhoP-activated genes as a readout of PhoP activity upon heterologous expression of PinT (Fig. 4A, *right*) in a *pinT* mutant strain in mildly acidic pH conditions. The strain carrying the PinT expression plasmid exhibited reduced levels of the *ugtL* coding region as compared to the one with the empty vector (Fig. 4A), most likely as a consequence of PinT promoting destabilization of *ugtL* mRNA by inhibiting its translation and the reduction of UgtL-mediated activation of PhoP resulting from it. Heterologous expression of PinT also reduced the abundance of *ugtL* 5′ leader region as well as that of the PhoP-activated gene *pagC*, encoding the outer membrane protein PagC that promotes formation of outer membrane vesicles in acidic pH conditions (27, 28), but did not affect the levels of the PhoP-independent gene *ompC* (Fig. 4A). PagC protein amounts were higher in the *pinT* mutant strain than those in wild-type *S*. Typhimurium in mildly acidic pH conditions at 6 and 8 h, but not at 4 h (Fig. 4B), in agreement with PinT reaching maximal expression (Fig. 2A) and repressing UgtL (Fig. 2C) at later time points and the sRNA having a negative effect on *pagC* expression (Fig. 4A) by reducing UgtL-mediated activation of PhoP. In contrast, PagC levels were similar between wild-type *S*. Typhimurium and in the *pinT* mutant in low Mg^2+^ medium (Fig. S4), consistent with UgtL being required for PhoQ to activate PhoP (and then *pagC*) in acidic pH but not in response to low Mg^2+^ (14). PinT’s negative effect on PagC expression occurs through the repression of *ugtL* because *pinT* inactivation in a *S*. Typhimurium strain harboring the chromosomal *ugtLM1* mutation abolishing PinT repression (Fig. 3E and 4C) or in a *ugtL* mutant (Fig. S5) did not significantly impact PagC abundance.

The PhoP-P-to-PhoP ratio in mildly acidic pH was reduced 1.9-fold in the pPinT-carrying strain as compared to the strain with the empty vector, corresponding to 18% of total PhoP protein, similar to that observed for the *ugtL* mutant strain in which the UgtL-mediated enhancement of PhoP activity is abolished (Fig. 4D), as previously reported (14). This is consistent with the heterologous expression of PinT reducing the mRNA abundance of PhoP-dependent genes (Fig. 4A) and the *pinT* mutant strain showing increased levels of PhoP-activated PagC in the same growing conditions (Fig. 4B). Moreover, the PhoP-P-to-PhoP ratio was higher in the *pinT* mutant strain than in wild-type *S*. Typhimurium in mildly acidic pH conditions, the difference between the two strains being the largest at 6 and 8 h (Fig. 4E). This is in agreement with PinT expression peaking at later time points (Fig. 2A) and the difference in UgtL (Fig. 2C) and PagC (Fig. 4B) amounts between wild-type *S*. Typhimurium and *pinT* mutant strain being the most pronounced at these time points.

These data indicate that PinT reduces PhoP activity through the repression of *ugtL* in mildly acidic pH conditions.

### The PinT-*ugtL* interaction is conserved in *S. enterica*

**Fig 5:**
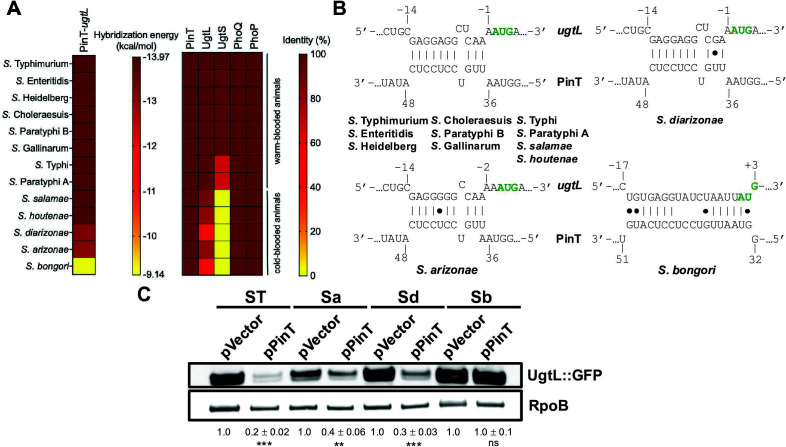
PinT-*ugtL* interaction is conserved in *Salmonella enterica*. (**A**) Heat map of PinT-*ugtL* base-pairing conservation and of PinT, UgtL, UgtS, PhoQ, and PhoP sequence identity among *Salmonella* species and serovars. Nucleotide sequences for PinT and deduced amino acid sequences for UgtL, UgtS, PhoQ, and PhoP proteins of *S. enterica* subsp. *enterica serovar* Enteritidis (P125109S; *S*. Enteritidis), *S. enterica* subsp. *enterica* serovar Heidelberg (41578; *S*. Heidelberg), *S. enterica* subsp. *enterica* serovar Choleraesuis (SC-B67; *S*. Choleraesuis), *S. enterica* subsp. *enterica* serovar Paratyphi B (SPB7; *S*. Paratyphi B), *S. enterica* subsp. *enterica* serovar Gallinarum (9184; *S*. Gallinarum), *S. enterica* subsp. *enterica* serovar Typhi (Ty2; *S*. Typhi), *S. enterica* subsp. *enterica* serovar Paratyphi A (ATCC11511; *S*. Paratyphi A), *S. enterica* subsp. *salamae* (Locarno; *S. salamae*), *S. enterica* subsp. *houtenae* (NCTC 7318; S. houtenae)*, S. enterica* subsp. *diarizonae* (SA20044251; *S. diarizonae*), *S. enterica* subsp. *arizonae* (RKS2980; *S. arizonae*), and *S. bongori* (NCTC12419) were compared to those of wild-type *S*. Typhimurium. Hybridization energy values determined by IntaRNA (26) for each PinT-*ugtL* duplex were plotted in a heat map. (**B**) *In silico* prediction of PinT-*ugtL* interaction in the *Salmonella* species and serovars from (**A**). Predictions were performed using the IntaRNA program (26). (**C**) Western blot analysis of crude extracts from *pinT* (HS1440) *S*. Typhimurium harboring plasmid pPinT or pVector (empty pUHE-21 vector) and pXG10sf-*ugtL-171* (*S*. Typhimurium; ST), pXG10sf-*ugtL-171Sa* (*S. arizonae*; Sa), pXG10sf-*ugtL-171Sd* (*S. diarizonae*; Sd), or pXG10sf-*ugtL-171Sb* (*S. bongori*; Sb) grown in N-minimal acidic pH (pH 4.9, 1 mM MgCl_2_) medium supplemented with 1 mM IPTG for 6 h. Samples were analyzed with antibodies directed to the GFP or RpoB proteins. Numbers below the blot indicate UgtL::GFP amounts relative to the pVector sample for each construct. Data are representative of three independent experiments (mean ± standard deviation), which gave similar results. **P < 0.01; ***P < 0.001, ns, not significant (*P* > 0.05), two-tailed unpaired *t* test with pPinT vs pVector for −182 and −171 GFP reporter constructs.

PinT is highly conserved across the *Salmonella* genus (≥ 98% shared identity in the nucleotide sequence), including in the nonpathogenic species *S. bongori* and the *S. enterica* subspecies *salamae*, *arizonae*, *houtenae*, and *diarizonae* (Fig. 5A), which are mostly associated with cold-blooded animals (29, 30). It was previously determined that the PhoP-dependent small protein UgtS that exerts negative feedback regulation of PhoP activity by antagonizing UgtL’s ability to promote PhoP activation has a narrow distribution across nontyphoidal *S. enterica* serovars infecting warm-blooded animals (Fig. 5A and [4]). We examined whether the PinT-mediated negative feedback of PhoP was restricted to the same serovars or if it was more widely distributed. We determined that the PinT-*ugtL* interaction is fully conserved in both nontyphoidal and typhoidal serovars infecting warm-blooded animals and in the *S. enterica* subspecies *salamae* and *houtenae* predominantly associated with cold-blooded animals (Fig. 5A and B). The PinT-*ugtL* duplex is slightly weakened in *S. enterica* subspecies *arizonae* (*S. arizonae*) and *diarizonae* (*S. diarizonae*) as compared to *S*. Typhimurium and the other species we predicted for it to be conserved, with single canonical A-U base pairs replaced with G•U wobble base pairs (Fig. 5B). These mutations did not abolish the PinT-mediated repression of *ugtL* when introduced in the *ugtL-171-gfp* reporter construct, thus suggesting that PinT represses *ugtL* in the two species (Fig. 5C). PinT-*ugtL* base pairing was predicted to be less stable in *S. bongori* as compared to *S*. Typhimurium, *S. arizonae,* and *S. diarizonae* (Fig. 5A and B), which is consistent with PinT failing to reduce UgtL::GFP abundance in a strain carrying an *S. bongori* variant of the *ugtL-171-gfp* reporter (Fig. 5C). These data suggest that the repression of *ugtL* by PinT does not occur in *S. bongori* and is rather restricted to *S. enterica*.

In summary, the broader distribution of PinT-*ugtL* interaction across the *Salmonella* genus as compared to that of UgtS suggests that the negative feedback regulation of PhoP activity via the post-transcriptional repression of *ugtL* by PinT benefits a larger set of *Salmonella* species and serovars than UgtL protein antagonization by UgtS.

## DISCUSSION

Despite the PhoP/PhoQ system being essential for *S*. Typhimurium’s survival inside macrophages by allowing the induction of the PhoP virulence program in response to acidic pH conditions (13), its activity must be finely regulated for the bacterium to cause disease. This is because constitutive *phoP* expression is detrimental for virulence (15, 16). We have now established that the PhoP-dependent sRNA PinT mediates post-transcriptional negative feedback control of PhoP/PhoQ activity by repressing the PhoP-activated enhancer of PhoQ autophosphorylation UgtL (Fig. 1). PinT reduces the abundance of UgtL protein in mildly acidic pH (Fig. 2B and C) by inhibiting the translation of *ugtL* mRNA through base pairing with its Shine-Dalgarno sequence (Fig. 3A, B, D and E). This results in decreased UgtL-mediated activation of PhoP (Fig. 4D and E) and, hence, of the genes it activates (Fig. 4A through C). The PinT-*ugtL* interaction is conserved in *S. enterica*, but not in the nonpathogenic species *S. bongori* (Fig. 5).

UgtL promotes positive feedback control of PhoP activity in mildly acidic pH conditions. That is, PhoP-P activates *ugtL* transcription, and UgtL then increases PhoP-P amounts by enhancing PhoQ autokinase activity, thus resulting in further PhoP activation (14). Failure to interrupt this UgtL-mediated PhoP activation loop could possibly impair *S*. Typhimurium’s ability to establish an infection as constitutive *phoP* expression attenuates virulence (15, 16). This is most likely reflected by the multiple layers of negative feedback regulation the *ugtL* gene is subjected to. First, the small protein UgtS antagonizes UgtL’s activity of promoting PhoQ autophosphorylation (thus leading to increased PhoP activation) in mildly acidic pH conditions through direct interaction with UgtL protein (4). UgtS is encoded in a PhoP-activated operon with UgtL (*ugtSugtL*) and thus promotes negative feedback regulation of PhoP activation (PhoP activates *ugtSugtL* transcription and UgtS in turn prevents the UgtL-mediated activation of PhoP). Second, we have now unveiled that the sRNA PinT represses *ugtL* translation (Fig. 3), thus providing negative post-transcriptional control of UgtL amounts (Fig. 2B and C). As *pinT* transcription is activated by PhoP (10), PinT is part of a negative feedback loop controlling PhoP activity (Fig. 4).

In addition to inhibiting UgtL through different mechanisms, UgtS and PinT describe distinct expression profiles and hence operate at different time points. That is, UgtS accumulates at early time points in mildly acidic pH conditions when it reduces the amounts of PhoP-P by impairing UgtL activity (4) while PinT levels peak at later time points (Fig. 2A) when it represses *ugtL* translation (Fig. 2B and C), thus resulting in decreased UgtL-mediated activation of PhoP (Fig. 4). The antagonization of UgtL activity by UgtS likely has a more immediate effect on PhoP activity than PinT-mediated reduction of UgtL amounts through translational repression of *ugtL* as it may take some time for UgtL abundance to decrease and then PhoP-P amounts to be reduced after PinT is transcribed and interacts with *ugtL* mRNA. It is then possible that UgtS and PinT are taking part in a two-stage inhibition of UgtL to control the level of PhoP activation, with the former regulator impairing UgtL’s activity at early time points of growth in mildly acidic pH conditions and the latter regulator further repressing UgtL by reducing its amounts at later time points when UgtS abundance decreases dramatically (4). As UgtL amounts exceed UgtS’ in later time points, given the increased abundance of the *ugtSugtL_-171_* transcript isoform lacking the *ugtS* Shine-Dalgarno sequence, and then solely allowing *ugtL* translation, and the low abundance of the *ugtSugtL_-182_* mRNA permitting *ugtS* translation (4), UgtS may not be able to keep up with antagonizing UgtL. PinT would then maintain PhoP activity in a range allowing optimal induction of the PhoP virulence program in response to mildly acidic pH conditions by inhibiting *ugtL* translation from both *ugtSugtL_-182_* and *ugtSugtL_-171_* transcripts (Fig. 1 and 3D).

The control of the activity or amounts of transcriptional regulators by sRNAs encoded in their regulon is a common feedback regulation feature of stress response pathways. For example, the EnvZ/OmpR-activated sRNAs OmrA/B directly repress *ompR* translation and reduce *ompRenvZ* mRNA abundance in response to osmotic stress in *E. coli* (31, 32). While this regulation decreases the abundance of the response regulator OmpR, it does not impact the amounts of its active form (OmpR-P) nor the expression of the OmpR-activated *ompF* and *ompC* genes whose promoters are less sensitive to changes in OmpR levels (32). However, it reduces OmrA/B expression, thereby allowing the two sRNAs to restrict their own synthesis. Moreover, the sRNA MicF represses its own repressor, the transcriptional regulator Lrp, by directly inhibiting the translation of *lrp* mRNA, thus forming a positive feedforward loop promoting its own expression and restricting the regulation of Lrp regulon, including the *lrp* gene (33). Finally, the Fur-repressed sRNA RyhB promotes its own expression during iron starvation by repressing the translation of a small ORF required for the *fur* gene to be expressed (34). These examples of sRNAs exerting feedback control of their cognate transcriptional regulators differ from the PinT-mediated regulation of PhoP activity through the repression of UgtL, in that they involve direct post-transcriptional regulation of regulator genes.

To the best of our knowledge, we report the first case of sRNA-mediated negative feedback control of a regulator that occurs through the downregulation of a factor promoting its activation. Although *ugtL* is expressed under both low Mg^2+^ (17) and mildly acidic pH conditions (4, 14) in a PhoP-dependent manner, along with PinT (Fig. 2A and [11]), the gene is required for PhoP activation in the latter condition only (14). Therefore, having PinT reducing PhoP activity by repressing *ugtL* translation instead of targeting the *phoPphoQ* transcript most likely renders its negative feedback action on PhoP specific to mildly acidic pH conditions, as suggested by *pinT* inactivation not impacting the levels of PagC in low Mg^2+^ conditions (Fig. S4). Because the *ugtL* gene was reported to mediate *S*. Typhimurium’s resistance to the cationic antimicrobial peptides magainin-2 and polymyxin B in low Mg^2+^ (35), PinT may regulate this activity through the repression of *ugtL* under this condition. Reducing the amounts of UgtL rather than PhoP abundance may also allow the bacterium to achieve the proper regulatory output as the latter regulation would be expected to impair the induction of the PhoP virulence program more drastically than the former.

Like UgtL, the *E. coli*-specific protein SafA promotes PhoQ autophosphorylation during acidic pH conditions (36–38). Unlike *ugtL*, the *safA* gene is not under transcriptional control of the PhoP/PhoQ system, but of the *E. coli*-specific EvgA/EvgS pathway that is activated by mildly acidic pH conditions. While no post-transcriptional regulator has been identified for *safA* yet, one cannot exclude a PhoP- or EvgA-dependent sRNA to exert negative feedback control of SafA by downregulating its expression, in a manner analogous to PinT repressing *ugtL*.

In addition to PinT and UgtS acting as negative regulators of UgtL, *S*. Typhimurium relies on the regulatory activity of the small protein MgrB to exert negative feedback control of PhoP activity (39–41). The *mgrB* gene is transcriptionally activated by PhoP, and MgrB reduces PhoP activity by inhibiting PhoQ’s autokinase activity (39). Unlike PinT and UgtS that are *Salmonella*-specific (4, 10), MgrB is conserved across Enterobacterales (39), which suggests that its regulatory action on PhoP benefits a broad range of species occupying various niches and having diverse lifestyles. While UgtS is only found in nontyphoidal *Salmonella* serovars that infect warm-blooded animals, in all of which it is presumed to antagonize UgtL (4), PinT-mediated repression of *ugtL* is predicted to occur in both typhoidal and pathogenic nontyphoidal *Salmonella* serovars associated with either warm- or cold-blooded animals (Fig. 5). That the PinT-mediated post-transcriptional repression of *ugtL* is more widespread than UgtL antagonization by UgtS suggests it benefits a larger set of *Salmonella* serovars occupying diverse habitats. The predicted erosion of PinT-*ugtL* interaction (Fig. 5B) and subsequent loss of PinT-mediated repression of *ugtL* (Fig. 5C) observed in the nonpathogenic species *S. bongori* is consistent with PhoQ being insensitive to mildly acidic pH conditions in this bacterium (13) in which UgtL probably serves a distinct function than *S*. Typhimurium UgtL as the two proteins only share 56% identity (Fig. 5A). This may also be an indication that the primary purpose of *ugtL* repression by PinT is to hamper the UgtL-dependent activation of PhoP in mildly acidic pH conditions rather than any yet-to-be-characterized activity UgtL may exhibit in other PhoP-inducing conditions leading to *ugtL* transcription.

The identification of PinT as an additional regulator of *ugtL* represents an extra layer of reciprocal regulation between the ancestral PhoP/PhoQ system and horizontally acquired regulatory factors. That is, PhoP activates the horizontally acquired gene loci *ugtSugtL* (4) and *pinT* (10). In turn, UgtL increases PhoP activity (14), the process of which is impaired by UgtS (4) and PinT (Fig. 4). Moreover, PhoP promotes the expression of the horizontally acquired regulator SsrB (42), which in turn activates *phoP* and relieves the silencing of *ugtL* transcription by H-NS (18). Such reciprocal regulatory circuits may allow *S*. Typhimurium to coordinate the induction of its virulence program during infection.

The validation of *ugtL* as a PinT target gene expands the regulon and further broadens the physiological role of the sRNA. In addition to promoting the transition from the SPI-1 invasion program to the SPI-2 intracellular replication program by downregulating the transcriptional activators of SPI-1 genes HilA and RtsA (11) as well as the SopE and SopE2 effectors allowing entry into epithelial cells (10), and delaying SteC-mediated actin rearrangements by repressing the *steC* gene (12), we now report that PinT controls the level of induction of the PhoP virulence program by decreasing the amounts of its activator UgtL (Fig. 1). Because PhoP directly activates the transcription of the *ssrB* gene, encoding the SPI-2 activator SsrB (42), PinT-mediated inhibition of UgtL is also expected to negatively impact the induction of SPI-2 genes.

PinT represses *ugtL* through a mechanism similar to that of its previously characterized mRNA targets, that is through base pairing in the vicinity of the Shine-Dalgarno sequence and start codon to repress translation (10–12). PinT-mediated repression of *ugtL* is likely Hfq-dependent, given that Hfq is required for PinT-*ugtL in vitro* duplex formation (Fig. 3B) and for PinT’s regulatory activity (12) as well as *in vivo* accumulation (43, 44).

Cumulatively, our findings establish that pathogens rely on multiple layers of negative feedback to control the activation of their virulence programs. Those operate through distinct mechanisms and at specific times, thereby allowing optimal and timely induction of virulence genes.

## MATERIALS AND METHODS

### Bacterial strains, plasmids, primers, and growth conditions

Bacterial strains and plasmids used in this study are listed in Table S1, and oligonucleotide sequences are presented in S2 Table. Single-gene knockouts and deletions were carried out as described (45). Mutations generated with this approach were subsequently moved into clean genetic backgrounds via phage P22-mediated transduction, as described (46). Bacteria were grown at 37°C in Luria-Bertani broth (LB) or N-minimal media pH 4.9 or 7.7 (47) supplemented with 0.1% casamino acids, 38 mM glycerol, and the indicated concentrations of MgCl_2_. *E. coli* DH5α was used as the host for the preparation of plasmid DNA. Ampicillin was used at 50 µg/mL, kanamycin at 50 µg/mL, and chloramphenicol at 20 µg/mL.

### Strain construction

Mutant strains were constructed using the one-step inactivation method (45) with pKD3 or pKD4 plasmid DNA as the template.

To construct the *pinT::Km^R^* strain (HS1440), a PCR product generated with primers W4361-W4362 using the pKD4 plasmid as a template was integrated into wild-type *S*. Typhimurium strain 14028 s via the one-step inactivation method (45) using the pKD46 plasmid. The insertion was confirmed by PCR with HUSA065–HUSA066 primers.

To construct the *ugtL::Cm^R^* strain (HS1547), a PCR product generated with primers W4463–W4094 using the pKD3 plasmid as a template was integrated into wild-type *S*. Typhimurium strain 14028 s via the one-step inactivation method (45) using the pKD46 plasmid. The insertion was confirmed by PCR with HUSA067–HUSA068 primers.

To generate the *ugtLM1* strain (AMPB002), a first PCR fragment was generated with primers HUSA697 and HUS698 using the pSLC-242 plasmid (48) as a template. The resulting PCR product was then integrated into the chromosome of wild-type *S. enterica* (14028 s) via the one-step inactivation method (45) using the pKD46 plasmid. Recombinant cells with the insertion were selected on LB supplemented with 20 µg/mL chloramphenicol at 30°C. This insertion was then replaced through a second pKD46-mediated recombination of pre-annealed HUSA699 and HUSA700 primers into the chromosome. Cells were recovered for 3 h as described (48) and selected on N-minimal medium agar plates (49) containing 50 µM glutamate, 50 µM histidine, 50 µM leucine, 100 µM methionine, 100 µM glutamine, 10 mM MgCl_2_, and 30 mM rhamnose as the sole carbon source. The allele replacement was confirmed by PCR with primers HUSA701 and HUSA702 and then by DNA sequencing.

### Construction of plasmids

To construct pUHE-*pinT*, primers HUSA035–HUSA036 were used to amplify *pinT* (+1 to +114, relative to *pinT* TSS) using *S*. Typhimurium strain 14028s genomic DNA as template. The resulting PCR product was digested with EcoRI and HindIII and ligated into the pUHE-21 plasmid (50) digested with the same restriction enzymes. The ligation reaction was transformed into DH5α cells by electroporation. The identity of the *pinT* insert was verified by DNA sequencing using primers HUSA007–HUSA008.

To construct pUHE-*pinTM1*, primers HUSA279–HUSA036 were used to amplify *pinTM1* (+1 to +114, relative to *pinT* TSS) harboring C45G mutation using *S*. Typhimurium strain 14028s genomic DNA as template. The resulting PCR product was digested with EcoRI and HindIII and ligated into the pUHE-21 plasmid (50) digested with the same restriction enzymes. The ligation reaction was transformed into DH5α cells by electroporation. The identity of the *pinTM1* insert was verified by DNA sequencing using primers HUSA007–HUSA008.

To construct pXG10sf-*ugtL-171*, primers HUSA180–HUSA182 were used to amplify the *ugtL _-171 to +390_* region (relative to the *ugtL* ATG start codon) using *S*. Typhimurium strain 14028s genomic DNA as template. The resulting product was then digested with NheI and NsiI and ligated into pXG10sf digested with the same enzymes. The ligation reaction was transformed into DH5α cells by electroporation. The identity of the *ugtL* insert was verified by DNA sequencing using primer HUSA058.

To construct pXG10sf-*ugtLM1-171*, primers HUSA180–HUSA318 and HUSA317–HUSA182 were used to amplify complementary fragments that carry the G-11C (M1) mutation and harbor NsiI and NheI sites. The two DNA fragments were annealed and amplified by PCR to obtain a *ugtLM1_-171+390_* fragment with NsiI and NheI sites. The resulting fragment was digested with NsiI and NheI and then ligated in the pXG10sf vector digested with the same restriction enzymes. The ligation reaction was transformed into DH5α cells by electroporation. The identity of the *ugtLM1_-171+390_* insert was verified by DNA sequencing using primer HUSA058.

To construct pXG10sf-*ugtLSd-171* (*ugtL* reporter with *S. diarizonae*’s PinT interaction site mutation), primers HUSA180–HUSA704 and HUSA703–HUSA182 were used to amplify complementary fragments harboring NsiI and NheI sites. The two DNA fragments were annealed and amplified by PCR to obtain a *ugtLSd_-171+390_* fragment with NsiI and NheI sites. The resulting fragment was digested with NsiI and NheI and then ligated in the pXG10sf vector digested with the same restriction enzymes. The ligation reaction was transformed into DH5α cells by electroporation. The identity of the *ugtLSd_-171+390_* insert was verified by DNA sequencing using primer HUSA058.

To construct pXG10sf-*ugtLSa-171* (*ugtL* reporter with *S. arizonae*’s PinT interaction site mutation), primers HUSA180–HUSA706 and HUSA705–HUSA182 were used to amplify complementary fragments harboring NsiI and NheI sites. The two DNA fragments were annealed and amplified by PCR to obtain a *ugtLSa_-171+390_* fragment with NsiI and NheI sites. The resulting fragment was digested with NsiI and NheI and then ligated in the pXG10sf vector digested with the same restriction enzymes. The ligation reaction was transformed into DH5α cells by electroporation. The identity of the *ugtLSa_-171+390_* insert was verified by DNA sequencing using primer HUSA058.

To construct pXG10sf-*ugtLSb-171* (*ugtL* reporter with *S. bongori*’s PinT interaction site mutations), primers HUSA180–HUSA708 and HUSA707–HUSA182 were used to amplify complementary fragments harboring NsiI and NheI sites. The two DNA fragments were annealed and amplified by PCR to obtain a *ugtLSb_-171+390_* fragment with NsiI and NheI sites. The resulting fragment was digested with NsiI and NheI and then ligated in the pXG10sf vector digested with the same restriction enzymes. The ligation reaction was transformed into DH5α cells by electroporation. The identity of the *ugtLSb_-171+390_* insert was verified by DNA sequencing using primer HUSA058.

To construct pXG10sf-*ugtL-182*, primers W3503-HUSA182 were used to amplify the *ugtL _-182 to +390_* region (relative to the *ugtL* ATG start codon) using *S*. Typhimurium strain 14028 s genomic DNA as template. The resulting product was then digested with NheI and NsiI and ligated into pXG10sf digested with the same enzymes. The ligation reaction was transformed into DH5α cells by electroporation. The identity of the *ugtL* insert was verified by DNA sequencing using primer HUSA058.

### Western blot assay

Overnight cultures of cells grown in N-minimal medium (pH 7.7) (47) supplemented with 10 mM of MgCl_2_ were diluted 1:50 in mildly acidic pH N-minimal medium (pH 4.9, 1 mM MgCl_2_) or low Mg^2+^ N-minimal medium (pH 7.7, 10 µM MgCl_2_), and cultures were grown for the indicated time points. Media were supplemented with 50 µg/mL ampicillin for strains carrying pUHE-based vectors and 20 µg/mL chloramphenicol for strains harboring pXG10sf constructs. To extract total proteins, cells were precipitated with trichloroacetic acid (5% total volume) and washed twice with ice-cold 80% acetone. Cell pellets were resuspended in NuPAGE LDS sample buffer (Thermo Fisher Scientific) and normalized according to the OD_600_. Protein samples were run on NuPAGE 4%–12% Bis-Tris Mini Protein gels (Thermo Fisher Scientific) and transferred to a nitrocellulose membrane by wet transfer using a Mini Blot Module device (Thermo Fisher Scientific) at 10 V for 75 min in 1× NuPAGE transfer buffer (Thermo Fisher Scientific) supplemented with 10% methanol. Membranes were blocked with 5% milk solution in TBS supplemented with 0.1% Tween (TBST) for 30 min and then probed with 1:5,000 dilutions in 2.5% milk solution in PBS of mouse anti-GFP (Sigma), mouse anti-FLAG (Sigma), or mouse anti-RpoB (BioLegend). Secondary horseradish peroxidase-conjugated anti-mouse (Promega) or anti-rabbit (Cytiva) was used at 1:5,000 dilution. Blots were developed using the Cytiva Amersham ECL Western blotting detection reagents (Cytiva) or SuperSignal West Femto Maximum Sensitivity substrate (Thermo Fisher Scientific). Images were acquired with ChemiDoc imager (Bio-Rad) and quantified using ImageLab software (Bio-Rad) by normalizing protein levels to the loading control RpoB.

### Northern blot assay

Overnight cultures of cells grown in N-minimal medium (pH 7.7) (47) supplemented with 10 mM of MgCl_2_ were diluted 1:50 in mildly acidic pH N-minimal medium (pH 4.9, 1 mM MgCl_2_), and cultures were grown for the indicated time points. Media were supplemented with 50 µg/mL ampicillin for strains carrying pUHE plasmids and 20 µg/mL chloramphenicol for strains harboring pXG10sf constructs. Total RNA was extracted using the hot phenol procedure, as previously described (51). Five micrograms of total RNA samples was run on 6% Novex TBE-Urea gel (Thermo Fisher Scientific) and transferred to Amersham Hybond-N + nylon membrane (Cytiva) at 250 mA for 2 h at 4°C in 0.5× TBE using the Mini-Trans Blot system (Bio-Rad). The membrane was then cross-linked at 1,200 J and pre-incubated for 30 min at 42°C in Church buffer (52). DNA probes complementary to PinT (HUSA404 or HUSA709) or 5S ribosomal RNA (HUSA405) were 5′-^32^P-radiolabeled with 20 µCi of [γ-^32^P]-ATP (Revvity) using T4-PNK polynucleotide kinase (New England Biolabs) according to the manufacturer’s directions. Hybridization was carried out overnight with the 5′-^32^P-radiolabeled DNA probes in Church buffer. The membrane was washed twice for 5 min with 2× SSC, 0.1% SDS and then twice for 15 min with 0.1× SSC, 0.1% SDS and exposed with a phosphor screen. Images were acquired with Amersham Typhoon IP imager (Cytiva) and quantified using ImageLab software (Bio-Rad) by normalizing RNA levels to the loading control 5S.

### Enzymatic probing assay

DNA templates harboring a T7 promoter sequence with the sequence of either *ugtL-171_-171+66_* (position relative to the *ugtL* ATG start codon) or *pinT* downstream were generated by PCR using primers HUSA312–HUSA313 (*ugtL-171_-171+66_*) and HUSA321–HUSA322 (*pinT*). Both *ugtL-171_-171+66_* and PinT RNAs were then synthesized by *in vitro* transcription from the generated DNA templates using the Megascript T7 Transcription Kit (Thermo Fisher Scientific) according to the manufacturer’s directions, and *ugtL-171_-171+66_* was subsequently 5′-^32^P-radiolabeled with 20 µCi of [γ-^32^P]-ATP (Revvity) using T4-PNK polynucleotide kinase (New England Biolabs) according to the manufacturer’s directions. Enzymatic probing was performed as described before (19) with some modifications. Briefly, 5′-^32^P-radiolabeled *ugtL-171_-171+66_* RNA (trace amounts) was denatured 1 min at 90°C and snap-cooled on ice for 5 min. Yeast RNA (Thermo Fisher Scientific) was then added (0.1 mg/mL final) along with RNA Structure Buffer (Thermo Fisher Scientific). PinT and/or purified Hfq protein were added, and the reaction mixtures were incubated for 10 min at 37°C. RNase T1 (0.1U; Thermo Fisher Scientific) digestion was then carried out for 2 min at 37°C. The reactions were stopped with 88 µL of stop solution (50 mM Tris-Cl pH 8.0, 0.1% SDS) and purified with 100 µL of phenol:chloroform 5:1 (Sigma), and then ethanol-precipitated. Sample pellets were resuspended with 10 µL of water and 10 µL of urea loading buffer (8 M urea, 1 mM EDTA pH 8.0, 0.09 M Tris, 0.09 M borate, 0.1% SDS, 0.05% bromophenol blue, and 0.05% xylene cyanol). Alkaline ladder was generated by incubating 5′-^32^P-radiolabeled *ugtL-171_-171+66_* RNA for 5 min at 90°C in 1× Alkaline Hydrolysis Buffer (Thermo Fisher Scientific). Guanine ladder was generated by first incubating 5′-^32^P-radiolabeled *ugtL-171_-171+66_* RNA for 1 min at 90°C in 1× RNA sequence buffer (Thermo Fisher Scientific) and then 5 min with 0.1 U of RNase T1 (Thermo Fisher Scientific) at 37°C. Both alkaline and guanine ladder reactions were stopped by the addition of urea loading buffer. Samples were run on 8% polyacrylamide, 7 M urea gel, which was then exposed with a phosphor screen. Gel images were acquired using Amersham Typhoon IP imager (Cytiva).

### Quantitative RT-PCR

Overnight cultures of bacterial cells grown in N-minimal medium (pH 7.7) (47) supplemented with 10 mM of MgCl_2_ were diluted 1:50 in mildly acidic pH N-minimal medium (pH 4.9, 1 mM MgCl_2_), and cells were grown for the indicated time points. Total RNA was extracted using the hot phenol procedure as previously described (51) and was treated with TURBO DNase (Thermo Fisher Scientific) before proceeding to cDNA synthesis using the SuperScript IV VILO Master Mix (Thermo Fisher Scientific). Transcript abundance was measured by qRT-PCR using Fast SYBR Green Master Mix (Applied Biosystems) in the QuantStudio 3 Real-Time PCR system (Applied Biosystems). Relative abundance values for each mRNA were determined using a standard curve obtained from serial dilutions of genomic DNA from wild-type *S*. Typhimurium (strain 14028 s). The mRNA abundance of *rrs* (primers HUSA003–HUSA004), *ugtL* 5′ leader (primers HUSA375–HUSA376), *ugtL* (primers HUSA043–HUSA044), *pagC* (primers HUSA183–HUSA184), and *ompC* (primers HUSA379–HUSA380) was measured, and values were normalized to *rrs* abundance.

### *In vivo* detection of phosphorylated PhoP

Overnight cultures of bacterial cells grown in N-minimal medium (pH 7.7) (47) supplemented with 10 mM of MgCl_2_ were diluted 1:50 in mildly acidic pH N-minimal medium (pH 4.9, 1 mM MgCl_2_), and cells were grown for the indicated time points. Cell extracts were prepared as previously described (53) with some modifications. Briefly, cells were spun-down according to OD_600_ (500 µL of cells spun-down for an OD_600_ of 0.5) and resuspended in 66 µL of ice-cold 1 M formic acid by trituration and vortexing. Twenty-six microliters of 4× LDS-sample buffer (Thermo Fisher Scientific) (1× final) supplemented with 4% β-mercaptoethanol (1% final) and 12 µL of 5 N NaOH were then added. Samples were run at 4°C on SuperSep Phos-tag (50 µmol/L), 12.5% gels (FUJIFILM Wako Pure Chemical Corporation) in standard running buffer (0.1% SDS, 25 mM, 192 mM glycine) at 150V for 4 h. Gels were washed for 15 min in 1× NuPAGE transfer buffer (Thermo Fisher Scientific) supplemented with 20% methanol and 1 mM EDTA and then for 15 min in EDTA-free transfer buffer. Samples were transferred to a nitrocellulose membrane using the Mini Blot Module device (Thermo Fisher Scientific) at 15 V for 75 min in 1× NuPAGE transfer buffer supplemented with 20% methanol. Membranes were blocked with 5% milk solution in TBS with 0.1% Tween (TBST) for 1 h and then probed with rabbit anti-PhoP (Thermo Fisher Scientific) (1:2,000 dilution) or mouse anti-AtpB (Abcam) (1:5,000 dilution). Secondary horseradish peroxidase-conjugated anti-mouse (Promega) or anti-rabbit (Cytiva) was used at 1:5,000 dilution. Blots were developed using the SuperSignal West Femto Maximum Sensitivity reagents (Thermo Fisher Scientific). Images were acquired with ChemiDoc imager (Bio-Rad) at a resolution of 300 ppi and quantified using ImageLab software (Bio-Rad).

## SUPPLEMENTAL MATERIAL

Supplemental figures msphere.00720-25-s0001.pdf Figures S1 to S6.

Legends msphere.00720-25-s0002.docx Legends for the supplemental figures.

Table S1 msphere.00720-25-s0003.docx Strains and plasmids.

Table S2 msphere.00720-25-s0004.docx Oligonucleotides.

ASM does not own the copyrights to Supplemental Material that may be linked to, or accessed through, an article. The authors have granted ASM a non-exclusive, world-wide license to publish the Supplemental Material files. Please contact the corresponding author directly for reuse.
