# Supplementary figures and images for: Small RNA promotes negative feedback of the master virulence regulator PhoP by repressing the PhoQ sensor enhancer UgtL in acidic pH
Source: mSphere. 2025 Dec 9;11(1):e00720-25. doi: 10.1128/msphere.00720-25 (PMC12838224; doi:10.1128/msphere.00720-25)

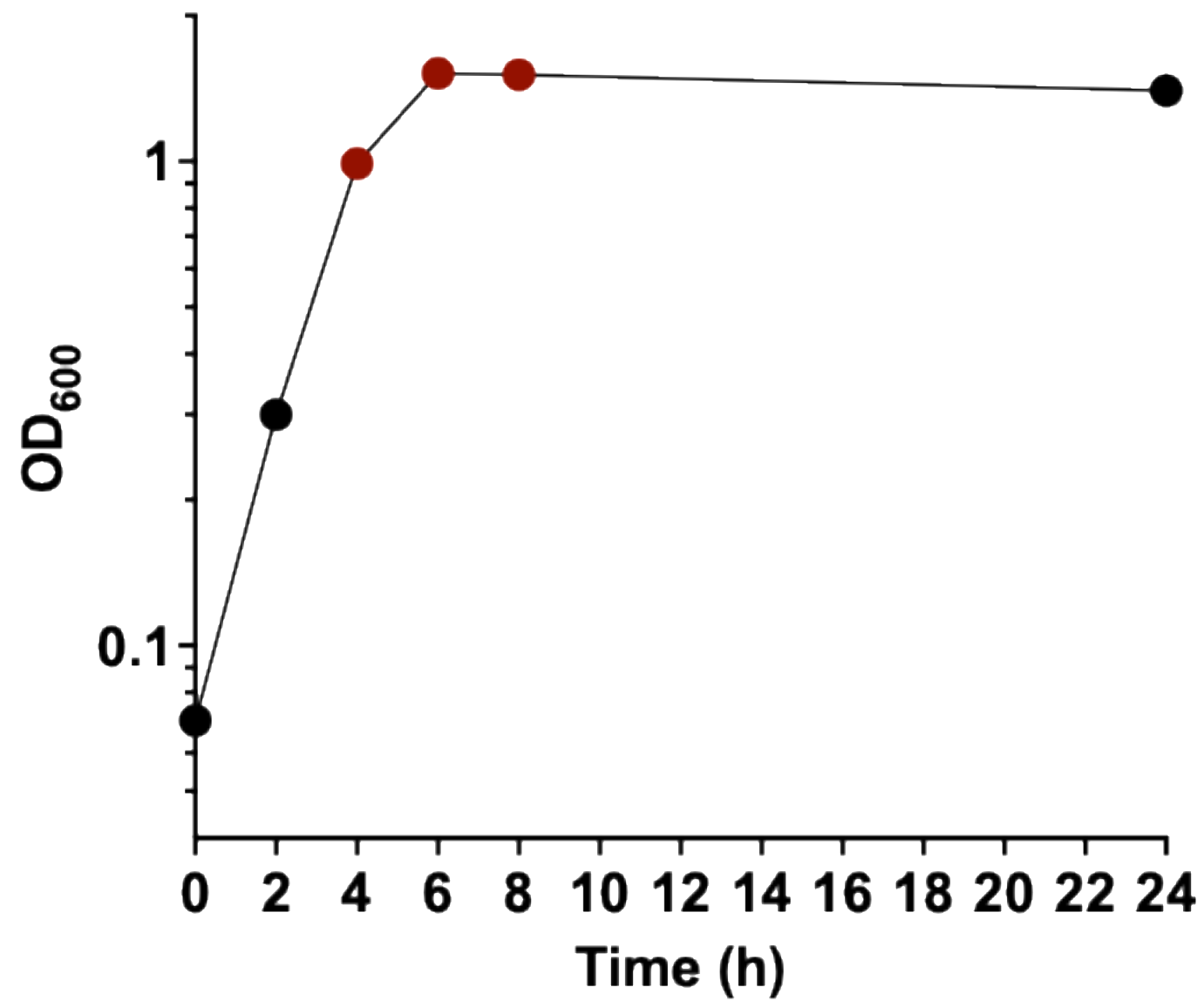

Fig. S1

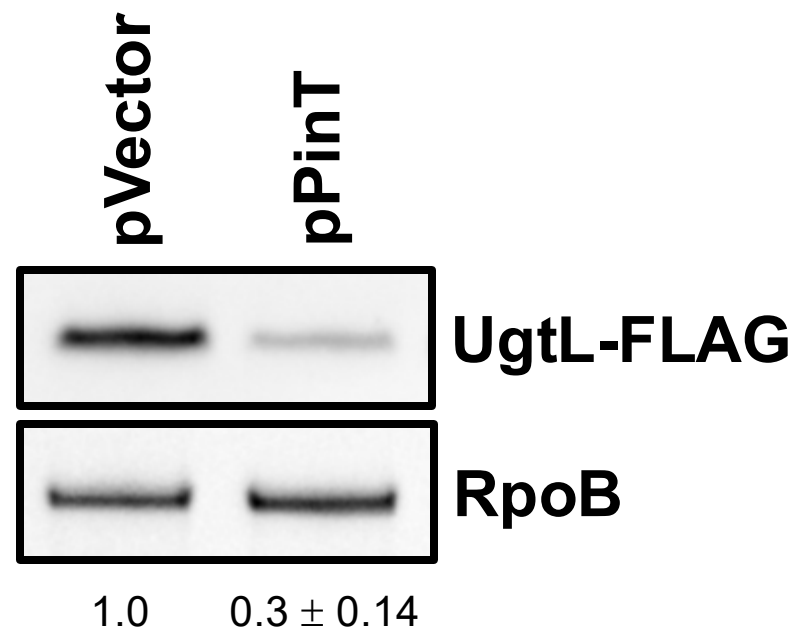

**Fig. S2**

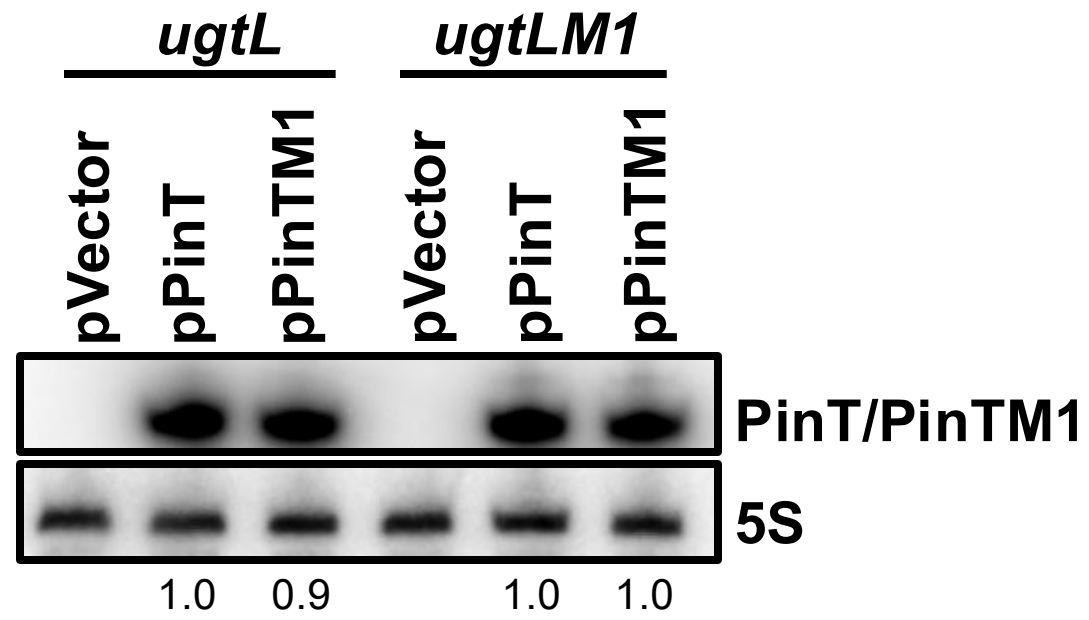

Fig. S3

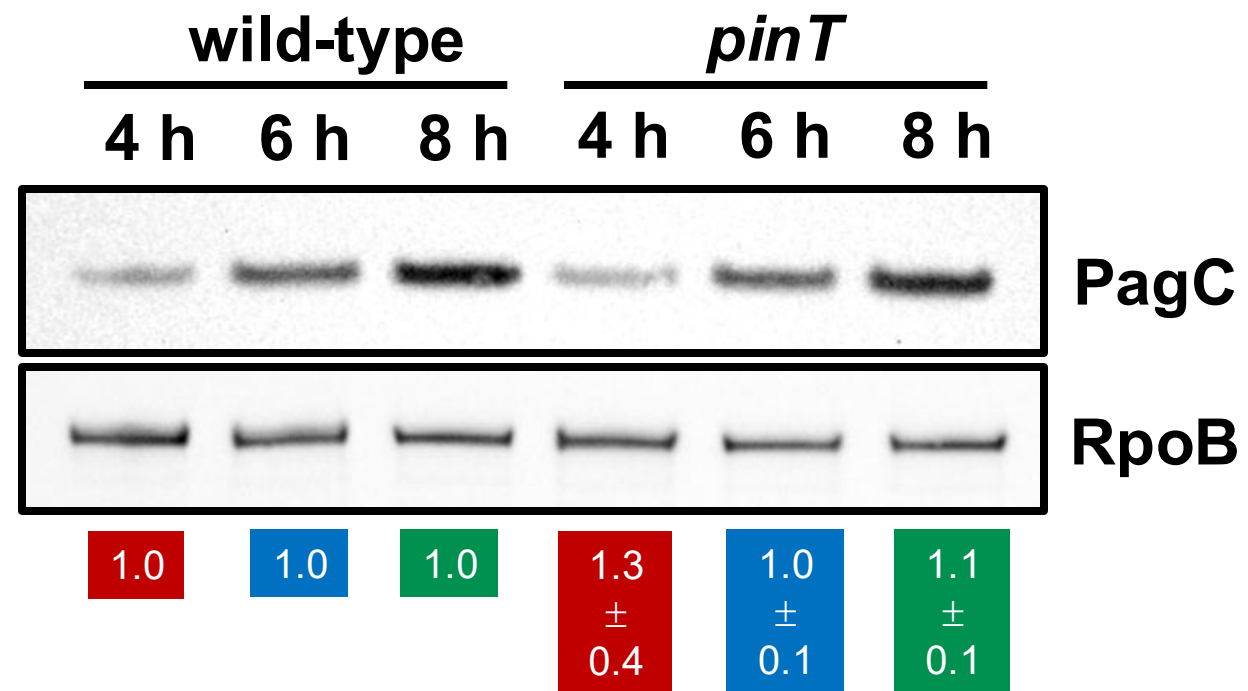

Fig. S4

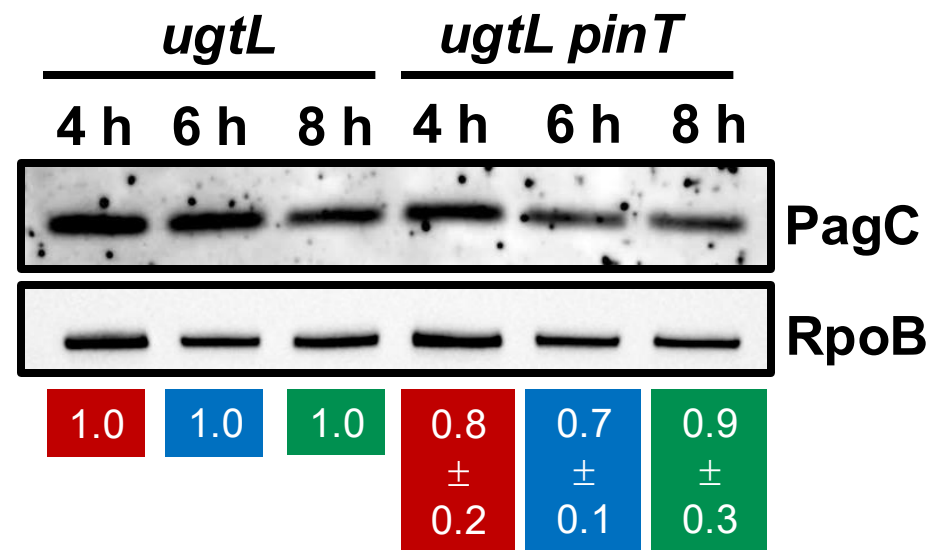

Fig. S5

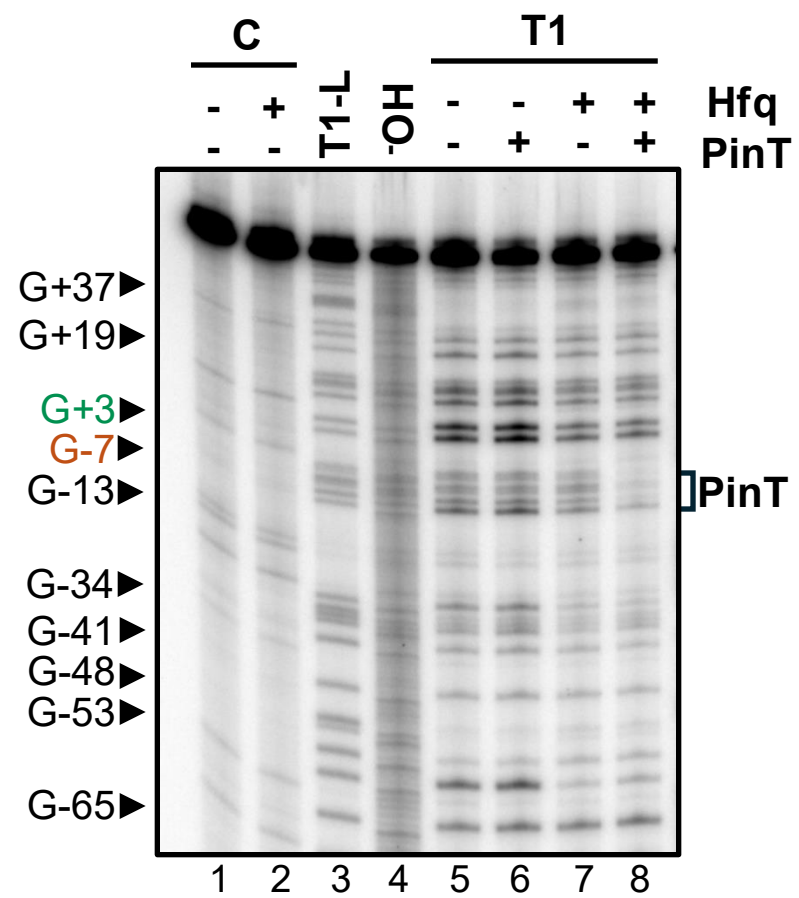

Fig. S6

Supplement: Supplemental figures — Figures S1 to S6. [file msphere.00720-25-s0001.pdf]
