# Supplementary material for: Small RNA promotes negative feedback of the master virulence regulator PhoP by repressing the PhoQ sensor enhancer UgtL in acidic pH
Source: mSphere. 2025 Dec 9;11(1):e00720-25. doi: 10.1128/msphere.00720-25 (PMC12838224; doi:10.1128/msphere.00720-25)
Supplement: Legends — for the supplemental figures. [file msphere.00720-25-s0002.docx]

**Supplemental figure legends**

**Fig. S1.** Representative growth curve of wild-type *S.* Typhimurium in N-minimal acidic pH (pH 4.9, 1 mM MgCl_2_) medium over a period of 24 h. 4, 6 and 8 h time points are indicated in red.

**Fig. S2.** Western blot analysis of extracts from *ugtL-FLAG pinT* (MIPR004) *S.* Typhimurium harboring plasmid pPinT or pVector (empty pUHE-21 vector) grown in N-minimal low Mg^2+^ medium (pH 7.7, 10 μM MgCl_2_) supplemented with 1 mM IPTG for 6 h. Samples were analyzed with antibodies directed to the FLAG epitope or the RpoB protein. Numbers below the blot indicate UgtL-FLAG levels relative to pVector sample. Data are representative of two independent experiments (mean±standard deviation), which gave similar results.

**Fig S3.** Northern blot analysis of PinT and PinTM1 abundance from total RNA extracts of *pinT* (HS1440) *S.* Typhimurium harboring pPinT, pPinTM1 or pVector (empty pUHE-21 vector), and pXG10sf-*ugtL-171* or pXG10sf-*ugtLM1-171*, grown in N-minimal acidic pH (pH 4.9, 1 mM MgCl_2_) medium supplemented with 1 mM IPTG for 6 h. Samples were analyzed with DNA probes complementary to PinT (HUSA709) or 5S ribosomal RNA (HUSA405). Numbers below the blot indicate PinTM1 levels relative to that of PinT for both *ugtL* and *ugtLM1* constructs.

**Fig S4.** Western blot analysis of crude extracts from wild-type (14028s) and *pinT* (HS1440) *S.* Typhimurium grown in N-minimal low Mg^2+^ medium (pH 7.7, 10 μM MgCl_2_) medium at the indicated time points. Samples were analyzed with antibodies directed to the PagC or RpoB proteins. Numbers below the blot indicate PagC levels in *pinT* background relative to wild-type background for a given time point depicted by color (red for 4 h; blue for 6 h; green for 8 h). Data are representative of two independent experiments (mean±standard deviation), which gave similar results.

**Fig S5.** Western blot analysis of crude extracts from *ugtL* (HS1547) and *ugtL* *pinT* (PABO220) *S.* Typhimurium grown in N-minimal acidic pH (pH 4.9, 1 mM MgCl_2_) medium at the indicated time points. Samples were analyzed with antibodies directed to the PagC or RpoB proteins. Numbers below the blot indicate PagC levels in *ugtL* *pinT* background relative to *ugtL* background for a given time point depicted by color (red for 4 h; blue for 6 h; green for 8 h). Data are representative of two independent experiments (mean±standard deviation), which gave similar results.

**Fig. S6.** Enzymatic probing analysis of PinT-*ugtL* interaction. Lower-contrast version of Fig. 3B.
