# Supplementary material for: Small RNA promotes negative feedback of the master virulence regulator PhoP by repressing the PhoQ sensor enhancer UgtL in acidic pH
Source: mSphere. 2025 Dec 9;11(1):e00720-25. doi: 10.1128/msphere.00720-25 (PMC12838224; doi:10.1128/msphere.00720-25)
Supplement: Table S1 — Strains and plasmids. [file msphere.00720-25-s0003.docx]

**S1 Table.** Bacterial strains and plasmids used in this study

| **Strains** | **Relevant characteristics** | **Source** |
| --- | --- | --- |
| ***Escherichia coli*** |  |  |
| DH5α | Host strain used for generation and propagation of plasmid constructs | (1) |
| ***Salmonella enterica* serovar Typhimurium** |  |  |
| 14028s | wild-type | (2) |
| HS1189 | *ugtL-FLAG*::*Cm^R^* | (3) |
| HS1440 | *pinT*::*Km^R^* | This study |
| HS1547 | *ugtL::Cm^R^* | This study |
| MIPR004 | *ugtL-FLAG*::*Cm^R^ pinT*::*Km^R^* | This study |
| PABO220 | *ugtL::Cm^R^ pinT*::*Km^R^* | This study |
| AMPB002 | *ugtLM1* | This study |
| AMPB003 | *ugtLM1 pinT*::*Km^R^* | This study |
| **Plasmids** | **Relevant characteristics** | **Source** |
| pKD3 | rep_R6Kg_ *Amp^R^* FRT *Cm^R^* FRT | (4) |
| pKD4 | rep_R6Kg_ *Amp^R^* FRT *Km^R^* FRT | (4) |
| pKD46 | rep_pSC101_^ts^ *Amp^R^* P_araBAD_-γβexo | (4) |
| pUHE-21 | rep_pMB1_ *lacI^q^* *Amp^R^* vector control | (5) |
| pUHE-*pinT* | rep_pMB1_ *lacI^q^* *Amp^R^* P_lac_-*pinT* | This study |
| pUHE-*pinTM1* | rep_pMB1_ *lacI^q^* *Amp^R^* P_lac_-*pinTM1* | This study |
| pXG10sf | rep _pSC101*_ *Cm^R^* P_LtetO_-*lacZ-gfp* | (6) |
| pXG10sf-*ugtL-182* | rep _pSC101*_ *Cm^R^* P_LtetO_-*ugtL-182-gfp* | This study |
| pXG10sf-*ugtL-171* | rep _pSC101*_ *Cm^R^* P_LtetO_-*ugtL-171-gfp* | This study |
| pXG10sf-*ugtL-171M1* | rep _pSC101*_ *Cm^R^* P_LtetO_-*ugtL-171M1-gfp* | This study |
| pXG10sf-*ugtL-171Sd* | rep _pSC101*_ *Cm^R^* P_LtetO_-*ugtL-171Sd-gfp* | This study |
| pXG10sf-*ugtL-171Sa* | rep _pSC101*_ *Cm^R^* P_LtetO_-*ugtL-171Sa-gfp* | This study |
| pXG10sf-*ugtL-171Sb* | rep _pSC101*_ *Cm^R^* P_LtetO_-*ugtL-171Sb-gfp* | This study |
| pSLC-242 | rep_R6Kγ_ *Amp^R^* FRT-(*Cm^R^* P*_rhaB_*-*relE*)-FRT | (7) |

**References**

1. Hanahan D. 1983. Studies on transformation of Escherichia coli with plasmids. J Mol Biol 166:557-80.

2. Fields PI, Swanson RV, Haidaris CG, Heffron F. 1986. Mutants of Salmonella typhimurium that cannot survive within the macrophage are avirulent. Proc Natl Acad Sci U S A 83:5189-93.

3. Salvail H, Choi J, Groisman EA. 2022. Differential synthesis of novel small protein times Salmonella virulence program. PLoS Genet 18:e1010074.

4. Datsenko KA, Wanner BL. 2000. One-step inactivation of chromosomal genes in Escherichia coli K-12 using PCR products. Proc Natl Acad Sci U S A 97:6640-5.

5. Soncini FC, Vescovi EG, Groisman EA. 1995. Transcriptional autoregulation of the Salmonella typhimurium phoPQ operon. J Bacteriol 177:4364-71.

6. Corcoran CP, Podkaminski D, Papenfort K, Urban JH, Hinton JC, Vogel J. 2012. Superfolder GFP reporters validate diverse new mRNA targets of the classic porin regulator, MicF RNA. Mol Microbiol 84:428-45.

7. Khetrapal V, Mehershahi K, Rafee S, Chen S, Lim CL, Chen SL. 2015. A set of powerful negative selection systems for unmodified Enterobacteriaceae. Nucleic Acids Res 43:e83.
