# Supplementary material for: Small RNA promotes negative feedback of the master virulence regulator PhoP by repressing the PhoQ sensor enhancer UgtL in acidic pH
Source: mSphere. 2025 Dec 9;11(1):e00720-25. doi: 10.1128/msphere.00720-25 (PMC12838224; doi:10.1128/msphere.00720-25)
Supplement: Table S2 — Oligonucleotides. [file msphere.00720-25-s0004.docx]

**S2 Table.** Oligonucleotides sequences used in this study

| **Name** | **Sequence (5′ -> 3′)** | **Purpose** | **Source** |
| --- | --- | --- | --- |
| HUSA003 | CCAGCAGCCGCGGTAAT | *rrs* qRT-PCR | This study |
| HUSA004 | TTTACGCCCAGTAATTCCGATT | *rrs* qRT-PCR | This study |
| HUSA007 | caattgtgagcggataacaatttc | Sequencing of pUHE-21 inserts | This study |
| HUSA008 | aatccagatggagttctgagg | Sequencing of pUHE-21 inserts | This study |
| HUSA035 | CAGTTGAATTCAGTAACGGATTACTTTGTGGTGTAG | Cloning of *pinT* | This study |
| HUSA036 | GCATCAAGCTTGTCTGTTAATTATTACAGAGAGAG | Cloning of *pinT* | This study |
| HUSA043 | CGGTATCATCGCAGGGTTTAT | *ugtL* qRT-PCR | This study |
| HUSA044 | ACGGGCGTGAAGAAACAT | *ugtL* qRT-PCR | This study |
| HUSA058 | TGGGATATATCAACGGTGGT | Sequencing of pXG10sf inserts | This study |
| HUSA065 | TTGTGAGTGAACCAAATCTG | *pinT::Kan^R^* verification | This study |
| HUSA066 | CACCATGCGAGGTCTCTT | *pinT::Kan^R^* verification | This study |
| HUSA067 | GTAGCTTTAGTCATGCCCACGCCTC | *ugtL::Cm^R^* verification | This study |
| HUSA068 | CACTGCAGTTGTGCAAACAC | *ugtL::Cm^R^* verification | This study |
| HUSA180 | GTTTTATGCATAACAACAATGAGATGTTTAG | Cloning of *ugtL* from -171 position relative to *ugtL* ATG start codon | This study |
| HUSA182 | GTTTTTGCTAGCTGAAGAAACATCCTGTG | Cloning of *ugtL* ending at +390 position relative to *ugtL* ATG start codon | This study |
| HUSA183 | AAAAGATTAAATCGGAGCGGGA | *pagC* qRT-PCR | This study |
| HUSA184 | TGACGCTCCATCCGCAATA | *pagC* qRT-PCR | This study |
| HUSA279 | CAGTTGAATTCAGTAACGGATTACTTTGTGGTGTAGCGTAACGGTAATTGTCCTCGTCATATTTG | Cloning of *pinTM1* | This study |
| HUSA312 | TGTAATACGACTCACTATAGGAACAACAATGAGATGTTTAG | Generation of DNA template for *ugtL-171+66* *in vitro* transcription | This study |
| HUSA313 | CCATTCTGACTGCAAAATGCCCCAG | Generation of DNA template for *ugtL-171+66* *in vitro* transcription | This study |
| HUSA317 | GATAAAATTATAAAAACCTGCGACGAGGCTCAAAATGAAGAAATCAGATGGTG | Cloning of *ugtLM1* | This study |
| HUSA318 | CACCATCTGATTTCTTCATTTTGAGCCTCGTCGCAGGTTTTTATAATTTTATC | Cloning of *ugtLM1* | This study |
| HUSA321 | TGTAATACGACTCACTATAGGAGTAACGGATTACTTTGTGGTGTAG | Generation of DNA template for PinT *in vitro* transcription | This study |
| HUSA322 | AAAAAGCGGCAGACTACGCTGCCGCA | Generation of DNA template for PinT *in vitro* transcription | This study |
| HUSA375 | TTTAGCGGTAGGGCAGAAG | *ugtL* 5’ leader qRT-PCR | This study |
| HUSA376 | GAAACAAAGCCGTCAGCTAAT | *ugtL* 5’ leader qRT-PCR | This study |
| HUSA379 | CCGACTGGTTAATGAGGGTT | *ompC* qRT-PCR | This study |
| HUSA380 | GGTACCAGGAGGGACAGTA | *ompC* qRT-PCR | This study |
| HUSA404 | TATGAGGAGGACAATTACCG | PinT DNA probe for Northern blot | This study |
| HUSA405 | CTACGGCGTTTCACTTCTGAGTTC | 5S DNA probe for Northern blot | This study |
| HUSA697 | GACGGCTTTGTTTCCAGTTGGGCGATAAAATTATAAAAACgtgtaggctggagctgcttc | Generation of *ugtLM1* strain | This study |
| HUSA698 | AGGATGCTGTCTTTTCGTGAATTTCACCATCTGATTTCTTcatatgaatatcctccttag | Generation of *ugtLM1* strain | This study |
| HUSA699 | GACGGCTTTGTTTCCAGTTGGGCGATAAAATTATAAAAACCTGCGACGAGGCTCAAAATGAAGAAATCAGATGGTGAAATTCACGAAAAGACAGCATCCT | Generation of *ugtLM1* strain | This study |
| HUSA700 | AGGATGCTGTCTTTTCGTGAATTTCACCATCTGATTTCTTCATTTTGAGCCTCGTCGCAGGTTTTTATAATTTTATCGCCCAACTGGAAACAAAGCCGTC | Generation of *ugtLM1* strain | This study |
| HUSA701 | GTAGGGCAGAAGGCCAATAC | Sequencing primer for *ugtLM1* allele insertion confirmation | This study |
| HUSA702 | CAGCAATAATAGCCGTCCAC | Sequencing primer for *ugtLM1* allele insertion confirmation | This study |
| HUSA703 | GATAAAATTATAAAAACCTGCGAGGAGGCTCGAAATGAAGAAATCAGATGGTG | Cloning of *ugtLSd* | This study |
| HUSA704 | CACCATCTGATTTCTTCATTTCGAGCCTCCTCGCAGGTTTTTATAATTTTATC | Cloning of *ugtLSd* | This study |
| HUSA705 | GATAAAATTATAAAAACCTGCGAGGGGGCTCAAAATGAAGAAATCAGATGGTG | Cloning of *ugtLSa* | This study |
| HUSA706 | CACCATCTGATTTCTTCATTTTGAGCCCCCTCGCAGGTTTTTATAATTTTATC | Cloning of *ugtLSa* | This study |
| HUSA707 | GATAAAATTATAAAAACCTGCGAGGTATCTAATTATGAAGAAATCAGATGGTG | Cloning of *ugtLSb* | This study |
| HUSA708 | CACCATCTGATTTCTTCATAATTAGATACCTCGCAGGTTTTTATAATTTTATC | Cloning of *ugtLSb* | This study |
| HUSA709 | GAGGACAATTACCGTTACGCTACACCACAAAG | PinT DNA probe for Northern blot (anneals upstream of PinTM1 mutation) | This study |
| W3503 | GTTTTATGCATATTATTAGGCTAACAACAATGAG | Cloning of *ugtL* from -182 position relative to *ugtL* ATG start codon | This study |
| W4361 | CAAGGTTATTAATACCATGATTTGACGATTGTTTGATTCGgtgtaggctggagctgcttc | Generation of *pinT::Kan^R^* strain | This study |
| W4362 | TCATTGTCTGTTAATTATTACAGAGAGAGTTAATTTATAAcatatgaatatcctccttag | Generation of *pinT::Kan^R^* strain | This study |
| W4463 | TTGGGCGATAAAATTATAAAAACCTGCGAGGAGGCTCAAAcatatgaatatcctccttag | Generation of *ugtL::Cm^R^* strain | This study |
| W4094 | CATAGCCATTATTCAGTAAGACCGCAGGTTGCAGCGGCGGgtgtaggctggagctgcttc | Generation of *ugtL::Cm^R^* strain | This study |
